# Cellular Therapy in Experimental Autoimmune Encephalomyelitis as an Adjuvant Treatment to Translate for Multiple Sclerosis

**DOI:** 10.3390/ijms25136996

**Published:** 2024-06-26

**Authors:** Maiara Carolina Perussolo, Bassam Felipe Mogharbel, Cláudia Sayuri Saçaki, Nádia Nascimento da Rosa, Ana Carolina Irioda, Nathalia Barth de Oliveira, Julia Maurer Appel, Larissa Lührs, Leanderson Franco Meira, Luiz Cesar Guarita-Souza, Seigo Nagashima, Caroline Busatta Vaz de Paula, Lucia de Noronha, Idiberto José Zotarelli-Filho, Eltyeb Abdelwahid, Katherine Athayde Teixeira de Carvalho

**Affiliations:** 1 Advanced Therapy and Cellular Biotechnology in Regenerative Medicine Department, The Pelé Pequeno Príncipe Research Institute, Child and Adolescent Health Research & Pequeno Príncipe Faculties, Curitiba P.O. Box 80240-020, Paraná, Brazil; perussolo10@gmail.com (M.C.P.); bassamfm@gmail.com (B.F.M.); claudiasacaki@gmail.com (C.S.S.); nadianr@gmail.com (N.N.d.R.); anairioda@gmail.com (A.C.I.); nathybarth03@gmail.com (N.B.d.O.); juliamappel@gmail.com (J.M.A.); luhrslarissa@gmail.com (L.L.); 2 Experimental Laboratory of the Institute of Biology and Health Sciences, Pontifical Catholic University of Paraná, Curitiba P.O. Box 80215-901, Paraná, Brazil; leandersonfm@gmail.com (L.F.M.); guaritasouzalc@hotmail.com (L.C.G.-S.); 3 Laboratory of Experimental Pathology, Graduate Program of Health Sciences, School of Medicine, Pontifical Catholic University of Paraná (PUCPR), Curitiba P.O. Box 80215-901, Paraná, Brazil; seigocap@gmail.com (S.N.); carolbvaz@gmail.com (C.B.V.d.P.); lnno.noronha@gmail.com (L.d.N.); 4 Postgraduate Program in Food, Nutrition and Food Engineering, Institute of Biosciences, Humanities and Exact Sciences (IBILCE), São Paulo State University (UNESP), São José do Rio Preto P.O. Box 15054-000, São Paulo, Brazil; zotarelli.filho@unesp.br; 5 Feinberg Cardiovascular Research Institute, Feinberg School of Medicine, Northwestern University, Chicago, IL 60611, USA; eltyeba@uic.edu

**Keywords:** human, Wharton’s jelly, mesenchymal stem cells, neural precursors, cellular therapy, experimental autoimmune encephalomyelitis, immunomodulatory, neuroprotection, multiple sclerosis, translation

## Abstract

This study aims to evaluate and compare cellular therapy with human Wharton’s jelly (WJ) mesenchymal stem cells (MSCs) and neural precursors (NPs) in experimental autoimmune encephalomyelitis (EAE), a preclinical model of Multiple Sclerosis. MSCs were isolated from WJ by an explant technique, differentiated to NPs, and characterized by cytometry and immunocytochemistry analysis after ethical approval. Forty-eight rats were EAE-induced by myelin basic protein and Freund’s complete adjuvant. Forty-eight hours later, the animals received intraperitoneal injections of 250 ng/dose of Bordetella pertussis toxin. Fourteen days later, the animals were divided into the following groups: a. non-induced, induced: b. Sham, c. WJ-MSCs, d. NPs, and e. WJ-MSCs plus NPs. 1 × 10^5^. Moreover, the cells were placed in a 10 µL solution and injected via a stereotaxic intracerebral ventricular injection. After ten days, the histopathological analysis for H&E, Luxol, interleukins, and CD4/CD8 was carried out. Statistical analyses demonstrated a higher frequency of clinical manifestation in the Sham group (15.66%) than in the other groups; less demyelination was seen in the treated groups than the Sham group (WJ-MSCs, *p* = 0.016; NPs, *p* = 0.010; WJ-MSCs + NPs, *p* = 0.000), and a lower cellular death rate was seen in the treated groups compared with the Sham group. A CD4/CD8 ratio of <1 showed no association with microglial activation (*p* = 0.366), astrocytes (*p* = 0.247), and cell death (*p* = 0.577) in WJ-MSCs. WJ-MSCs and NPs were immunomodulatory and neuroprotective in cellular therapy, which would be translated as an adjunct in demyelinating diseases.

## 1. Introduction

Among the main neurodegenerative diseases of the central nervous system (CNS), multiple sclerosis (MS) is a major cause of axonal degeneration related to autoimmune inflammatory demyelination, affecting approximately 2.8 million people worldwide [1,2]. According to the International Federation of MS (2020), the incidence of the disease has increased since 2013, when there were 2.3 million patients worldwide [1]. This increase was justified by improved diagnosis, patient support, and the ability to better account for MS cases. In Brazil, it is estimated that 40,000 people live with the disease, according to the data from the Brazilian MS Association [3]. The trigger factors responsible for developing the disease are still poorly understood. However, in recent years, environmental risk factors along with new genetic factors and the interaction between genetic and environmental factors have become the consensus in the literature as being the epigenetic causes of the disease [4,5].

The International MS Genetics Consortium analyzed genotypic data from individuals with MS and healthy controls, discovering genetic variants linked to autosomal susceptibility at the major histocompatibility complex and a variant on the X chromosome. These variants, combined with others found in the same study, even when not significant for the entire genome, may explain 48% of the hereditary factor of MS [6,7]. A study on mice using the EAE model observed epigenetic changes in oligodendrocytes. In these mice, the oligodendrocytes displayed increased chromatin accessibility, fewer histone marks, and modifications in chromatin structure in genes related to the immune system [8].

Different studies discuss and investigate the immunopathology and pathophysiology of the disease [9,10,11]. It is known that during the development of MS, clinical manifestations can range from fatigue to motor and cognitive disorders as the disease progresses over time [9]. The first clinically noticed episode is called Clinically Isolated Syndrome—which can involve the optic nerve, spinal cord, and brainstem, resolving over time and potentially evolving into one of the chronic forms of the disease, secondary progressive MS, or remaining relapsing–remitting MS—and it is the most common form of the disease [10,11]. About 10% of patients are diagnosed with primary progressive MS, which presents a progressive decline from the onset of the disease without periods of recovery [10].

The immune response in EAE involves a complex interplay between various immune cells, each contributing to the initiation, propagation, and resolution of the disease. CD4+ T cells, particularly Th1 and Th17 cells, play a central role in driving the inflammatory process, while regulatory T cells attempt to counterbalance this response. B cells, macrophages, microglia, dendritic cells, neutrophils, and NK cells also contribute to the pathology of EAE through mechanisms involving antigen presentation, cytokine production, and direct tissue damage. Understanding the roles of these immune cells in EAE provides valuable insights into the pathogenesis of multiple sclerosis and potential therapeutic targets [12,13]. Regarding the immunological profile of the CNS in patients with MS, it was established that various types of T helper cells (Th1, Th2, Th9, Th17, Th22), T cells (CD4+and CD8+), B cells, and macrophages are found, with their presence varying according to the stage of MS [12,13].

Disease-modifying therapies for MS mainly focus on immunomodulators such as dimethyl fumarate, β-INF, and glatiramer acetate [14]. However, available first-line treatments revolve around palliative actions, addressing existing symptoms, and promoting the reduction of relapses, proving effective in the most common form of the disease but falling short in progressive and chronic cases [15]. Given the need for advancements in treating MS, many studies focused on discovering new therapeutic technologies, mainly through animal models. Experimental autoimmune encephalomyelitis (EAE) is the most extensively used model for MS studies as it presents consistent similarities with the disease’s mechanism [16]. Axonal and neuronal degradation, demyelination, motor dysfunction in the lower limbs, blood–brain barrier disruption, infiltration of CD4+ and CD8+ T cells, activation of microglia and macrophages, and release of inflammatory cytokines are examples of clinical findings in EAE [17,18]. Clinical manifestations of EAE in rodents, such as paralysis and tail tone loss, are observable [16,19].

The induction of EAE is performed through the subcutaneous administration of an emulsion containing a synthetic peptide adjuvant derived from myelin protein, such as myelin oligodendrocyte glycoprotein (MOG) or myelin basic protein (MBP) [20]. MOG and MBP peptides are used in EAE induction because multiple sclerosis patients have high levels of anti-MOG and anti-MBP antibodies, justifying the use of these factors for EAE induction in animal models [21]. In addition to the classic induction peptides, Bordetella pertussis toxin (PTX) is used in the EAE model as an immune response adjuvant, allowing greater blood–brain barrier permeability and promoting a high recruitment of B cells, contributing to neuroinflammation [22,23].

One of the most investigated technologies currently involves cellular therapy. It was believed that mesenchymal stem cells (MSCs) could give rise to other cells through transdifferentiation, meaning they would alter their gene expression to that of a completely different cell lineage, originating distinct cell types [24,25]. Another proposal is that MSCs can fuse with a target adult cell, assuming the gene expression pattern of the cell they joined, which favors their use in in vivo cellular therapy [26]. Additionally, MSCs secrete various cytokines and growth factors, which can modify the microenvironment they are present in, inducing, for example, endogenous activity of tissue cell regeneration [23]. It is known that the ability of MSCs to induce paracrine effects stems from the production and excretion of extracellular vesicles called exosomes.

When compared to other MSCs, Wharton’s jelly derived MSCs (WJ-MSCs), despite being morphologically similar, present desirable advantages such as ease of collection, storage, and transport, an inexhaustible source, and the ability to migrate to injured sites. [26]. In addition to their migration, cell differentiation, and tissue regeneration capabilities, WJ-MSCs demonstrate significant importance in immune responses, modulating inflammatory responses [23,26].

Conversely, Dobuchak et al. (2022) recently demonstrated that neural precursor cells, a subpopulation from WJ-MSCs, could differentiate into neural cells such as Schwann cells and oligodendrocytes. These cells are hierarchically at the forefront of neuronal differentiation compared with MSCs. They are committed to being nervous cells, having the potential for better resolution regarding the injury of demyelination associated with unexpressed histocompatibility antigens D1 [27].

The latter characteristics were also present in WJ-MSCs. Due to these characteristics, both cellular types were good candidates for therapy in neurodegenerative diseases. [27,28]. Thus, this study aimed to evaluate the outcome and related histopathological findings after cellular therapies: neural precursors versus mesenchymal stem cells in EAE induced with MBP and PTX.

## 2. Results

### 2.1. Characterization of Wharton’s Jelly Mesenchymal Stem Cells and Neural Precursors

**Figure 1:**
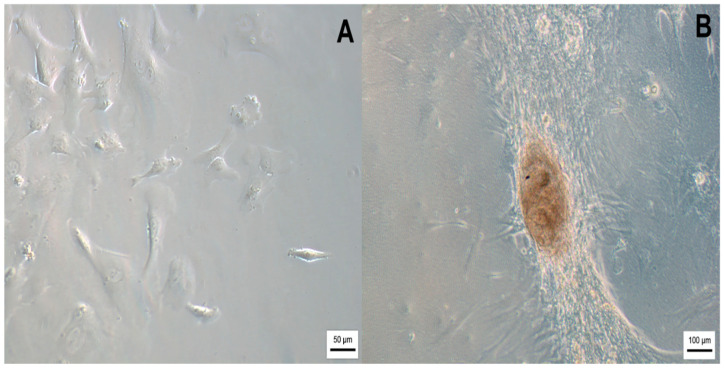
(**A**) WJ-MSCs; (**B**) Neurosphere after 14 days of neural precursors in culture. The images were obtained via optical microscopy (Carl Zeiss Microscopy, Zeiss, Jena, Germany).

After collection and isolation, WJ-MSCs were cultured until they showed 85% confluency. Fibroblastic morphology and adhesion to the polystyrene-cultured flasks were observed (Figure 1A). In addition, a fraction of these cells was differentiated into neural precursors after seeding in cultured flasks pre-coated by NFBX, as outlined by Stricker et al. (2021) and Dobuchak et al. (2022) [27,29].

The precursors were isolated after the formation of neurospheres (Figure 1B).

#### 2.1.1. Flow Cytometry Analysis

The results obtained from flow cytometry demonstrated a positive expression of specific cell surface markers of WJ-MSCs, such as CD13, CD73, CD90, and CD105. Hematopoietic markers CD34 and CD45 and the histocompatibility HLA-DR complex were not expressed. Moreover, Fluorescence Minus One (FMO) was performed as a technique control (Supplementary Figures S2–S4). The result of cell viability was approximately 98.80%.

#### 2.1.2. Trilineage Assay

**Figure 2:**
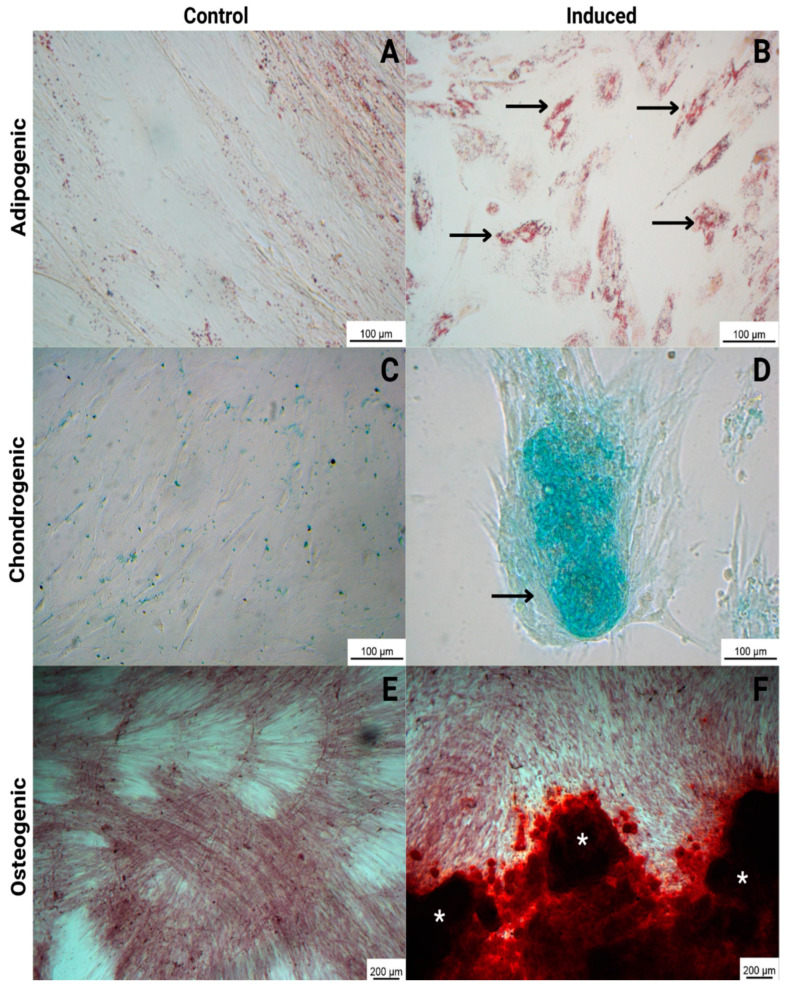
Trilineage differentiation of WJ-MSCs, comparing control (non-induced cells) and induced cells. Adipogenic: with lipid deposition in the cytoplasm of the cells detected by staining with oil red O (black arrows) (**B**); chondrogenic: with proteoglycan deposition detected by staining with alcian blue (black arrow) (**D**); and osteogenic: with calcium deposition detected by staining with alizarin red (white asterisks) (**F**). The control cells (non-induced) were negative for all staining procedures (**A**,**C**,**E**). The images were obtained via optical microscopy (Carl Zeiss Microscopy, Zeiss, Jena, Germany).

Mesenchymal stem cells were submitted to differentiate into adipogenic, osteogenic, and chondrogenic lineages to demonstrate pluripotency. The results demonstrated the ability of WJ-MSCs to differentiate in all tested lineages: adipogenic, with lipid deposition in the cytoplasm of the cells detected by staining with oil red O; osteogenic, with calcium deposition detected by staining with alizarin red; and chondrogenic, with proteoglycan deposition detected by staining with alcian blue (blue/green deposition). The control cells (non-induced) were negative for all staining procedures (Figure 2).

#### 2.1.3. Immunocytochemistry

**Figure 3:**
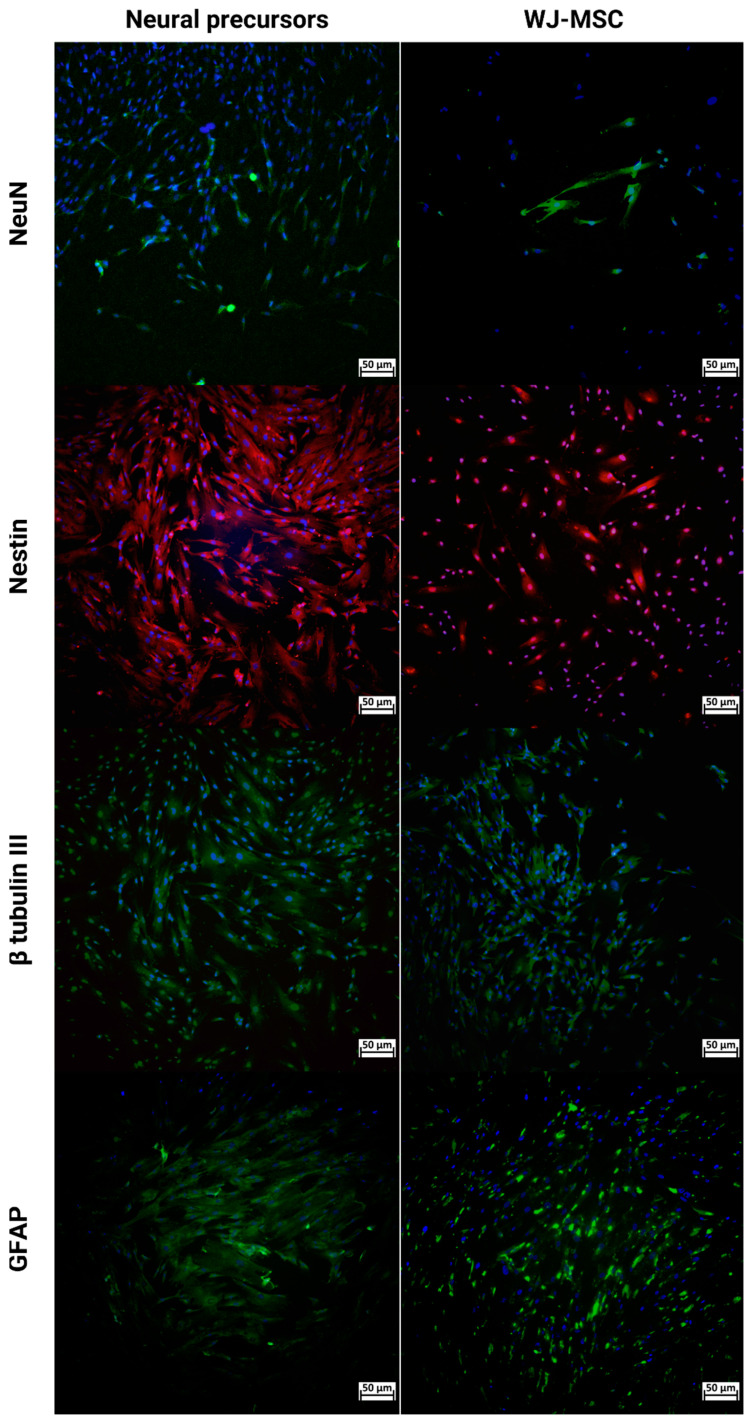
Immunocytochemistry of NPs and WJ-MSCs for NeuN (green), Nestin (red), β-tubulin III (green), and GFAP (green) markers. DAPI as a cell viability marker in blue. Images were obtained using a high-throughput microscope in Cell Analyzer 2000 (GE Healthcare^®^, Chicago, IL, USA). Wharton’s Jelly mesenchymal stem cells (WJ-MSCs) and neural precursors (NPs).

An immunocytochemistry assay was performed with WJ-MSCs and neural precursors (Figure 3). In both cases, cells expressed glial fibrillary acidic protein (GFAP), β-tubulin III, Nestin, and neuronal nuclear protein (NeuN).

**Figure 4:**
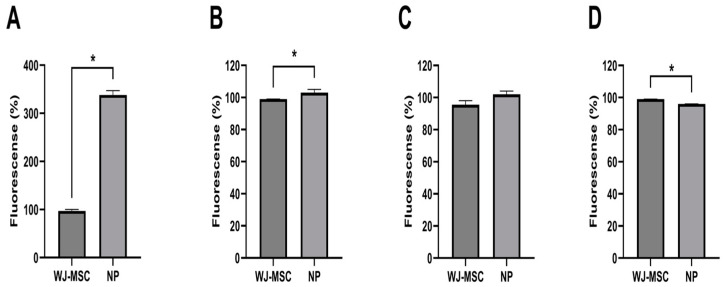
NeuN (**A**), Nestin (**B**), β-tubulin III (**C**), and GFAP (**D**) markers by immunocytochemistry. * *p* < 0.05 (n = 345).

Quantitative data regarding the analysis using the non-parametric Mann–Whitney test of the expression in the NPs and WJ-MSCs for NeuN, Nestin, and GFAP markers by immunocytochemistry demonstrated that there was a more significant statistical difference in neural precursors than WJ-MSCs (Figure 4).

### 2.2. Clinical Score Signs and Weight

**Figure 5:**
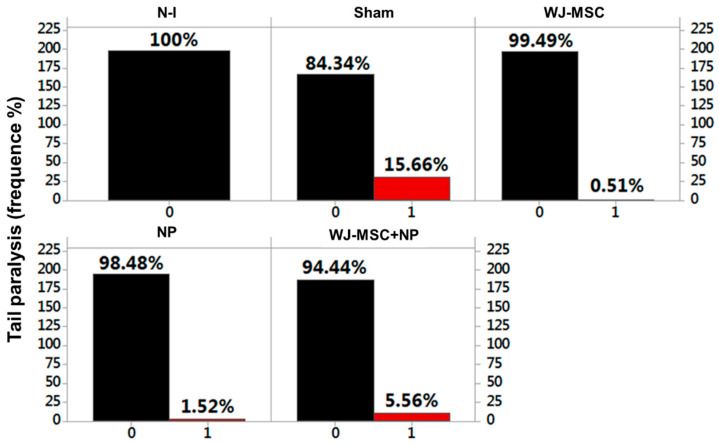
Frequency of clinical manifestation tail paralysis occurrence in the EAE model across the tested groups. Experimental autoimmune encephalomyelitis (EAE), non-induced group (N-I), Wharton’s jelly mesenchymal stem cells (WJ-MSCs) and neural precursors (NPs).

For 24 days, all the animals were analyzed by clinical manifestation and weight. The alteration “paralysis of tail” was considered as “absent” or “present,” resulting in a set of binary data (zero when absent; one when present). Thus, the data analysis revealed that the highest presence of a clinical manifestation was found in the Sham group (15.66%), followed by the WJ-MSC + NP group (5.56%), NP group (1.52%), and, lastly, the WJ-MSC group (0.51%) (Figure 5).

Using the predictive binary logistic regression analysis, it was observed that the animals that received MSCs, NPs, and MSC + NPs showed a statistically significant difference concerning the Sham group, with *p* > 0.05 for all the analyses, thus rejecting the null hypothesis. The relationships of MSCs v NPs and MSCs vs. MSCs + NPs also showed a statistically significant difference, with *p* = 0.902 > 0.05 and *p* = 0.809 > 0.05, respectively. The NPs vs. MSCs + NPs showed no statistical difference, with *p* = 0.034 < 0.05, not rejecting the null hypothesis (Supplementary Graphics SG1–SG3).

Using the Kruskal–Wallis non-parametric statistical analysis, it was observed that there was a significant statistical difference concerning the comparisons of the median weight values (g) between all variables (non-induced, Sham, MSCs, NPs, and MSCs + NPs), with *p* < 0.05 (rejecting the null hypothesis), except in the comparison between the Sham and MSCs groups (in red), with *p* = 0.363 > 0.05 (not rejecting the null hypothesis) (Table 1).

### 2.3. Histopathological Analysis

#### Morphological Analysis

**Figure 6:**
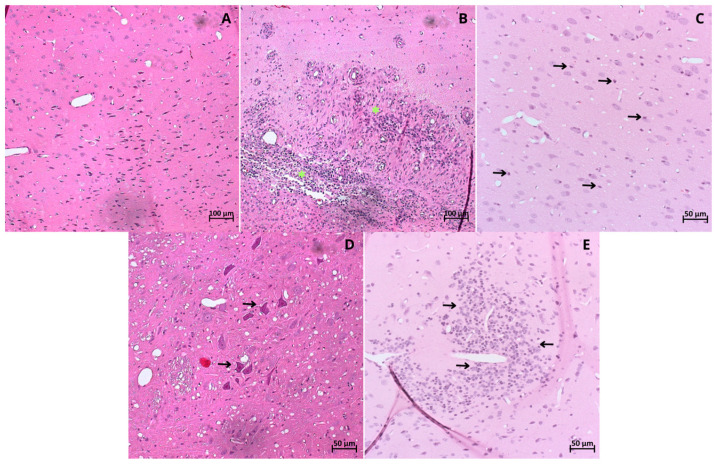
Brain tissue from (**A**) non-induced group; (**B**) Sham group: showing severe leukocyte infiltration (* in yellow); (**C**) WJ-MSCs therapy group: mild parenchymal infiltration (black arrows); (**D**) NPs therapy group: demonstrating cell death, pycnotic neurons (black arrows); (**E**) WJ-MSCs + NPs therapy group: showing astrocyte activation (black arrows). All stained with H&E obtained byoptical microscopy (Carl Zeiss Microscopy, Zeiss, Jena, Germany). Simulation of surgical stereotaxic treatment (Sham); Wharton’s jelly mesenchymal stem cells (WJ-MSCs), neural precursors (NPs).

The brain tissue collected post-euthanasia was used for histopathological analyses. H&E-stained slides were examined for findings including perivascular leukocyte infiltration, leukocytes within the parenchyma, microglial activation, astrocyte activation, and cell death, as observed through the visualization of pyknotic neurons (Supplementary Figure S3). In the non-induced animals, the tissue stained with H&E exhibited a homogeneous distribution of neurons and myelin (Figure 6).

**Figure 7:**
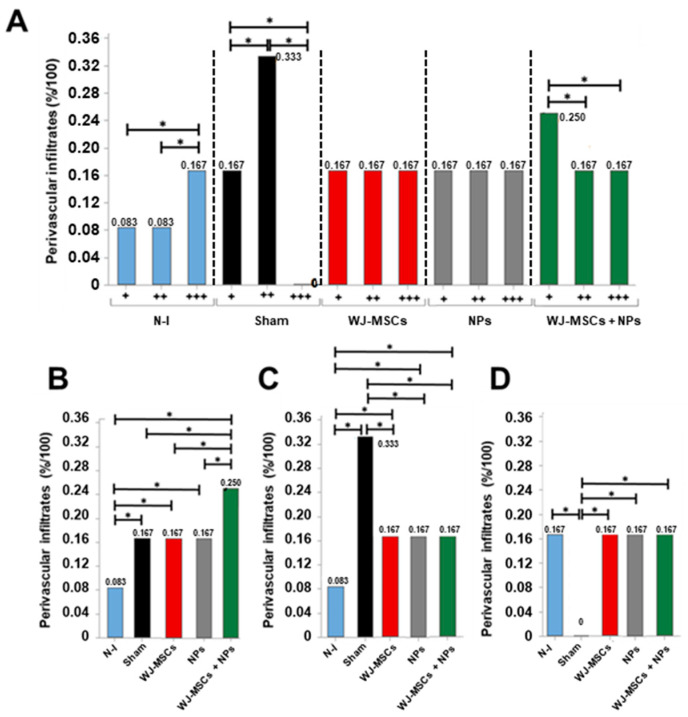
Histopathological analysis of the finding “Perivascular leukocytic infiltrates.” (**A**) Intragroup analysis of the categories “mild (+)”, “moderate (++)”, and “severe (+++)” (n = 8). (**B**) Between-group analysis for the “mild (+)” category; (**C**) between-group analysis for the “moderate (++)” category; (**D**) between-group analysis for the “severe (+++)” category. Results from Pearson’s Chi-squared test, considering *p* > 0.05 as statistically significant at the 95% confidence interval (*). Non-induced group (N-I), Wharton’s jelly mesenchymal stem cells (WJ-MSCs), and neural precursors (NPs).

Intra- and intergroup analyses were conducted for perivascular and parenchymal leukocyte infiltrates. Concerning perivascular infiltrates, the Sham group showed a higher incidence of medium-sized infiltrates (0.33%). In contrast, the WJ-MSCs and NPs groups displayed a homogeneous distribution across the three analyzed extents (0.167%), and the WJ-MSCs + NPs group demonstrated a higher incidence of small infiltrates (0.25%) (Figure 7A).

In intergroup comparisons, the groups induced in the EAE model exhibited a higher incidence of mild infiltrates than the non-induced group (Figure 7B). Regarding moderate infiltrates, all treated groups showed a reduction in incidence compared to the Sham group (Figure 7C). Severe perivascular infiltrates were observed with the exact same incidence in all groups except for the Sham group (Figure 7D). Photomicrography analysis is provided in Supplemental Figure S3.

**Figure 8:**
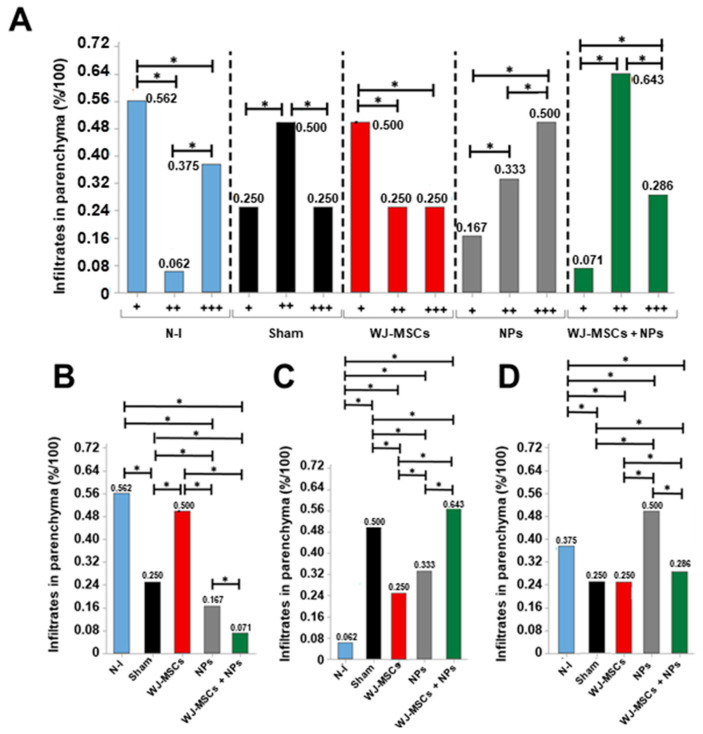
Histopathological analysis of the finding “Leukocytic infiltrates in parenchyma.” (**A**) Intragroup analysis of the categories “mild (+)”, “moderate (++)”, and “severe (+++)” (n = 8). (**B**) Between-group analysis for the “mild (+)” category; (**C**) between-group analysis for the “moderate (++)” category; (**D**) between-group analysis for the “severe (+++)” category. Results from Pearson’s Chi-squared test, considering *p* > 0.05 as statistically significant at the 95% confidence interval (*). Non-induced group (N-I), Wharton’s jelly mesenchymal stem cells (WJ-MSCs), and neural precursor (NPs).

Considering leukocytic infiltrates in the cerebral parenchyma, the Sham and WJ-MSCs + NPs group showed a higher incidence of moderate infiltrates (0.5% and 0.643%, respectively). The WJ-MSCs group exhibited a higher occurrence of mild infiltrates (0.5%), while the NP group showed severe infiltrates (0.5%) (Figure 8A). From intergroup analysis, it was observed that mild infiltrates differed among all groups, with a lower incidence in the NPs and WJ-MSCs + NP groups (Figure 8B). Concerning moderate infiltrates, all induced groups displayed increased incidence compared to the non-induced group.

However, the WJ-MSCs and NPs groups showed reduced incidence compared to the control (Figure 8C). The NPs group displayed increased values for severe infiltrates compared to all other groups (Figure 7D).

**Figure 9:**
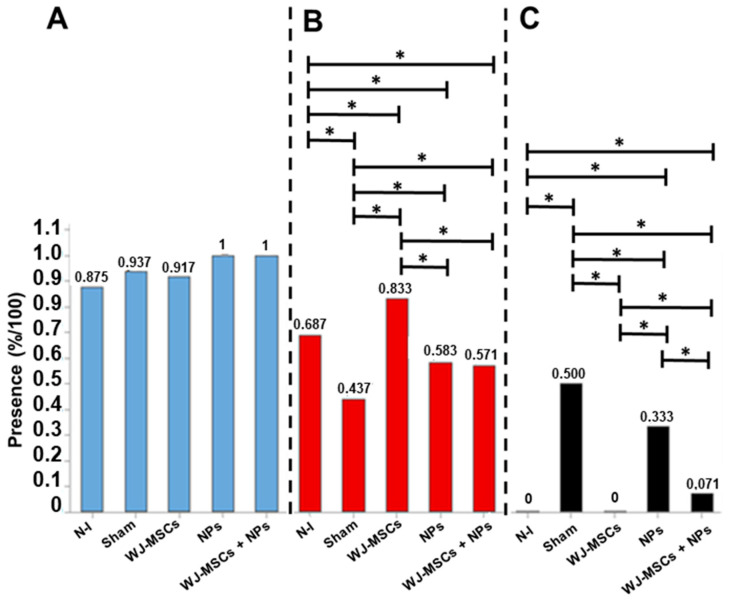
Histopathological analysis of microglial activation (n = 8) (**A**), astrocytic activation (n = 8) (**B**), and cell death (n = 8) (**C**). The results are from the Pearson’s Chi-squared test and consider *p* > 0.05 as statistically significant at the 95% confidence interval (*). Non-induced group (N-I), Wharton’s jelly mesenchymal stem cells (WJ-MSCs), and neural precursors (NPs).

Microglial activation was not statistically significantly different among the analyzed groups. However, the non-induced group showed the lowest activation level (0.875%) (Figure 9A).

For astrocytic activation, the results demonstrated an increase in activation in animals treated with WJ-MSCs compared to all other groups. Additionally, concerning astrocytes, the NPs and WJ-MSCs + NPs groups showed higher activation than the Sham group (Figure 9B). Regarding cell death, it was characterized by the presence of pyknotic cells, which was reduced in all treated groups compared to the Sham group.

When compared to other treatments, the WJ-MSCs group displayed the lowest cell death rate (Figure 9C).

**Figure 10:**
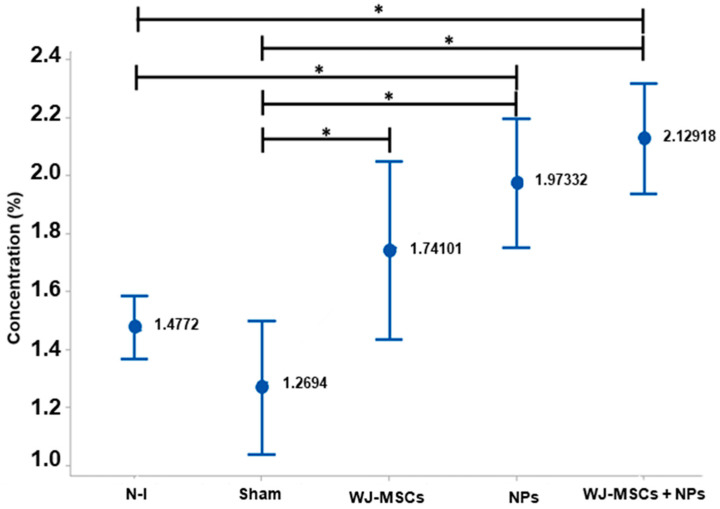
Histopathological analysis with Luxol Fast Blue in brain tissue (n = 8). Results from ANOVA analysis with Tukey’s post-test (mean ± standard error, and *p* < 0.05 (*)). Non-induced group (N-I), Wharton’s jelly mesenchymal stem cells (WJ-MSCs), and neural precursors (NPs).

Samples with Luxol were used to assess the presence of myelin in the tissue. The EAE model induced the formation of demyelinated plaques in the cerebral white matter, reducing the observed homogeneity. From the slides, it was possible to identify the amount of myelin in the tissue based on the Luxol concentration per area, allowing for the analysis of myelination. When compared, the Sham group differed from the WJ-MSCs group (*p* = 0.016), the NPs group (*p* = 0.010), and the WJ-MSCs + NPs group (*p* = 0.000). Additionally, the non-induced group showed a difference compared to the NP (*p* = 0.010) and WJ-MSCs + NPs groups (0.000) (Figure 10).

### 2.4. Histopathology and Immunohistochemistry (IHC) Analysis

#### 2.4.1. Cytokines

**Figure 11:**
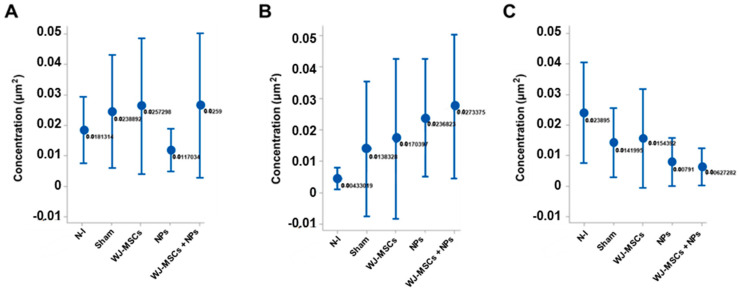
Cytokine concentration per square micrometer. (**A**) IL-4 concentration in the different analyzed groups, with no statistical difference (n = 8); (**B**) IFN-γ concentration among the groups, with no statistical difference (n = 8); (**C**) TNF-α concentration in the different groups, without statistically significant difference (n = 8). The results are from ANOVA analysis with Tukey’s post-test, displayed as mean ± standard error, and with *p* < 0.05. Non-induced group (N-I), Wharton’s jelly mesenchymal stem cells (WJ-MSCs), and neural precursors (NPs).

The cytokines IL-4 (Figure 11A), IFN-γ (Figure 11B), and TNF-α (Figure 11C) were measured via immunohistochemistry to evaluate tissue inflammation. None of the cytokines showed statistically significant differences.

#### 2.4.2. Membrane Cell Markers

**Figure 12:**
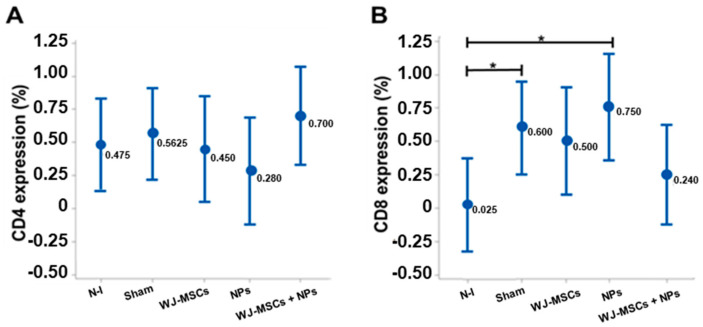
Lymphocytic markers in brain tissue. (**A**) Comparative quantification of CD4 cells between groups with no statistical difference (n = 8). (**B**) Comparative quantification of CD8 cells between groups (n = 8). Sham and NP groups had an increase in CD8 cells compared to the non-induced group. The results are from ANOVA analysis with Tukey’s post-test, displayed as mean ± standard error, and with *p* < 0.05 (*). Non-induced group (N-I), Wharton’s jelly mesenchymal stem cells (WJ-MSCs), and neural precursors (NPs).

IHC staining of lymphocyte markers CD4 and CD8 was performed. No statistical difference was found between the groups regarding the CD4 marker (Figure 12A). However, for the CD8 marker, the Sham and NP groups showed differences compared to the non-induced group (*p* = 0.026 and *p* = 0.007, respectively) (Figure 12B).

The CD4/CD8 ratio was calculated, and all animals with results ≤1 were selected for Pearson’s Chi-squared analysis regarding histopathological findings. This analysis revealed that WJ-MSCs could generate a lack of association (*p* > 0.05) concerning leukocytic infiltrates in the parenchyma, microglial, and astrocytic activation, as well as cell death, indicating the immunomodulatory potential of WJ-MSCs (Table 2). Considering the microglial and astrocytic activation and cell death findings, the WJ-MSCs + NPs group also did not show statistical association (*p* > 0.05), demonstrating the ability to modulate the immune response.

## 3. Discussion

This study aimed to evaluate the effects of cell therapy in a preclinical model of Experimental Autoimmune Encephalitis. The cells were of two types: stem cells derived from Wharton’s jelly and neural precursors. The NPs are a subfraction of MSCs, commissioned to be neurons. EAE presents itself as an alternative for a better understanding of the patterns of MS and their therapies as it is a model in which immunopathological features of MS are replicated and can be observed [30]. However, it is important to emphasize that animal models for multiple sclerosis, although capable of simulating some relevant aspects of the disease, are unable to reproduce the complex tissue, cellular, and biochemical alterations that occur in multiple sclerosis in humans. An example of this limitation is the induction of chronic lesions in EAE, involving B-cells and CD8 T-cells, which present in animal models in a milder form than in humans [31]. The choice of WJ-MSCs and NPs as a therapy in EAE stemmed from the well-known capacity of WJ-MSCs for immunomodulation and microenvironment modification. Additionally, NPs can differentiate into various neuronal types, such as oligodendrocytes [32].

When assessing the clinical manifestation of animals at this study stage, the Sham group demonstrated a higher frequency in the appearance of clinical signs (15.66%), justified by the induction without treatment. On the other hand, the WJ-MSC, NP, and WJ-MSC + NP groups showed a reduced frequency of clinical manifestation compared to the Sham group. In this induced EAE model, the immunopathological alterations are characteristic of inflammation, leading to demyelination lesions in the central nervous system (CNS) [32,33].

In the histopathological analysis, the EAE groups showed leukocyte infiltration, microglia, astrocyte activation, and pycnotic cells, indicating inflammatory findings. Microglial activation regularly occurs in EAE and MS. Chronic microglial inflammatory activity damages myelin and disrupts its axonal and synaptic activities [34]. Despite these detrimental effects, their potent phagocytic and tissue remodeling abilities support critical endogenous repair mechanisms [35].

However, understanding the role of microglia in these conditions is crucial. While under normal conditions, microglia display an anti-inflammatory phenotype (M2) and promote cerebral tissue homeostasis, in pathological conditions, like EAE and MS, the phenotype switches to pro-inflammatory (M1), triggering changes mediated by cytokines and reactive oxygen species, resulting in tissue damage [36]. Regarding the activation and proliferation of astrocytes, these cell types are related to myelin repair. In this study, WJ-MSCs induced higher astrocytic activation and prevented cell death in the treated animals. Through an in vitro examination, demonstrated that astrocyte subpopulations are involved in the remyelination processes of chronic lesions, providing cytokines that create a conducive microenvironment for myelin production [37]. In summary, while microglia are more directly involved in immune responses in the CNS, astrocytes have a broader role in supporting and maintaining the neural environment [38].

Additionally, when analyzing the myelin present in cerebral tissue, the results indicated a higher myelin concentration in all treated groups than in the Sham group. These findings were similar to those published by Brown et al. (2021), who demonstrated an increase in myelin in the brains of rats induced in the experimental autoimmune encephalomyelitis model 17 days after cellular therapy [30]. It suggests that WJ-MSCs and their subpopulations, like NPs, can protect nervous tissue from demyelination more than promoting remyelination, especially given the short post-transplantation period of only ten days [39,40]. This therapy could help prevent the demyelination process and potentially act as an adjunct treatment for MS.

The cytokines analyzed in this stage, IL-4, IFN-γ, and TNF-α, did not display significant statistical differences, which may be associated with the fact that IL-4 attenuates TNF-α production, as described by Gadani et al. (2012) [41]. The limitations that may have contributed to the absence of statistical differences between the groups in cytokines was the sample size.

An increase in IL-4 and INF-γ in the model-induced groups may also explain the low count of CD4 cells in cerebral tissue. It is known that in the presence of IL-4, CD4 T lymphocytes differentiate into Th1 cells, while in the presence of IL-12 and INF-γ, they differentiate into Th2 cells [42]. Another explanation for the lower number of CD4 cells in the treated groups may lie in the ability of WJ-MSCs to suppress the response of pro-inflammatory cells like CD4 T cells.

Luz-Crawford et al. (2013) demonstrated in a preclinical trial using the EAE model that mesenchymal stem cell transplantation suppressed the proliferation, activation, and differentiation of CD4 cells, promoting an immunosuppressive action [43]. As for the CD8 cell markers, there was an increase in the average presence of this cell type in all groups induced in the EAE model [43]. Camara et al. (2013) have already demonstrated that in Lewis rats, the induction of the EAE model and the intensity of the response to induction depends on the activation of CD8 cells, which may explain the results of the analysis in this study [44].

However, only the SHAM and NP groups showed statistical differences compared to the non-induced control in this study. CD4 and CD8 cells were used to assess proportion. The CD4/CD8 ratio refers to the proportion of two important T cell subpopulations in the immune system. In MS or its preclinical model, EAE, analyses of this ratio can be relevant to evaluate immune activity as it is characterized by an autoimmune response directed against the CNS. An increase in the CD4/CD8 ratio may indicate an increased activation of helper T cells (CD4), contributing to inflammation and damage to the nervous system [45].

Thus, the CD4/CD8 ratio analysis in the context of immunomodulation can evaluate how interventions or treatments affect T cell subpopulations in the immune system [45]. When using the CD4/CD8 ratio regarding histopathological findings, the results of this study pointed to the efficiency of WJ-MSCs in modulating the response of animals in the EAE model, which contributes to the study of this cell type and its subpopulations as adjunct therapies in the treatment of MS. This study had some limitations, such as the need for transplanted cell labeling, its sample size, and the short follow-up period.

The histopathological features of MS are the presence of demyelinated plaques in white matter and, to a lesser extent, in gray matter; the perivascular infiltration by lymphocytes, macrophages, and activated microglia; axonal transection and loss within demyelinated lesions; reactive gliosis or glial scarring, which is a common feature of chronic lesions and heterogeneous lesions in cellular composition; and the extent of remyelination. Regarding EAE, it is similarly demyelinated by lesions, primarily in the white matter of the CNS, with dense perivascular and parenchymal infiltration processes carried out by mononuclear cells (predominantly CD4+ T cells), axonal damage, and loss, although these are generally less pronounced than in MS. Moreover, the gliosis can be present but can vary depending on the EAE model and species used [46].

It is crucial to emphasize that, like other animal models, the pathological features can vary based on the species (e.g., mice and rats) and the myelin antigen used to induce EAE [46].

## 4. Conclusions

WJ-MSCs and NPs used as cell therapy in EAE can promote beneficial and relevant tissue changes along with treating symptoms. This type of therapy slowed down the brain demyelination process in the treated animals and reduced the rate of cell death compared to the control group, which was also known as the ‘Sham group’. The neuroprotective process occurred in all treated groups, indicating that both cell types can prevent disease progression when administered alone or in combination.

Interestingly, isolated WJ-MSCs demonstrated even more significant potential. They showed enhanced immunomodulation and neuroprotection, leading to greater astrocytic activation.

These findings strongly advocate for translating the neuroprotective and immunomodulatory effects of WJ-MSCs and NPs actions into future clinical research on MS. This is a crucial next step for enhancing our understanding of the potential treatments that can be used to manage this debilitating disease.

## 5. Materials and Methods

### 5.1. Animals

All procedures were approved by the Ethics Committee on the Use of Animals (CEUA) at the Pequeno Príncipe Complex, approval number 048-2020 (10 February 2020). The used animals were *Rattus norvegicus*, Lewis’s lineage, females aged 6 to 8 weeks, and weighing around 170 g. All the animals were kept in polypropylene boxes, receiving feeders and water ad libitum, with a 12 h light/dark cycle and an average temperature between 21 °C and 23 °C being maintained. The animals were acquired from Campinas University and kept at Pelé Pequeno Príncipe Research Institute.

### 5.2. Experimental Design

A total of 48 animals were used, which were randomly divided into five groups: (a) non-induced group (N-I) (n = 6); induced group: (b) simulation of surgical stereotaxic treatment (Sham) group (n = 6); (c) WJ-MSCs therapy group (n = 12); (d) NP therapy group (n = 12); and (e) WJ-MSCs + NPs therapy group (n = 12). The MSC stem cells were derived from Wharton’s jelly (WJ-MSCs) and the neural precursors (NPs), which were obtained from each sample through differentiation of the WJ-MSCs. These animals were maintained in groups of 2 or 3 animals per polypropylene cage.

### 5.3. EAE Induction

The animals were submitted with 200 µL of the emulsion containing 100 µg of the myelin oligodendrocyte glycoprotein (MPB) peptide (Sigma-Aldrich^®^, St. Louis, MO, USA) dissolved in 100 µL of 1% phosphate-buffered saline (PBS) (Sigma-Aldrich^®^, St. Louis, MO, USA) and emulsified in 100 µL of Freund’s Adjuvant Supplement (Thermo Fisher Scientific Inc., Waltham, MA, USA), which contained *Mycobacterium butyricum* to increase the immune response, for immunization that was subcutaneously injected on the dorsal region (100 µL) on both sides of the animals [16,33].

At the same time of induction and 48 h later, the animals received an intraperitoneal injection of 250 ng/dose of *Bordetella pertussis* toxin (Sigma-Aldrich^®^, St. Louis, MO, USA) [33]. The control group was submitted to the same procedures mentioned above but using PBS.

### 5.4. Clinical Signs of EAE

The severity of EAE was observed and classified daily according to the presence or absence of clinical signs. Animals with no clinical manifestation were considered zero (0), and those with tail paralysis were considered one (1). In addition to the clinical score, the animals were also evaluated daily for body weight.

### 5.5. Acquisition and Isolation of Wharton’s Jelly Mesenchymal Stem Cells

The umbilical cord samples used for this study were obtained after approval of the research project by the Human Ethical Committee of the Pequeno Príncipe Faculties, approval number 4.199.681, on 7 August 2020.

Two umbilical cords were collected from healthy mothers who had undergone prenatal care after signing the informed consented term. Following the collection, the samples were transported in 50 mL Falcon tubes containing 30 mL of 3000 UI/mL penicillin and 0.3 mg/mL streptomycin diluted in PBS (3% P/S PBS (Sigma-Aldrich®, San Luis, Missouri) at room temperature to the Pelé Pequeno Príncipe Research Institute and processed within 4 h after birth.

The isolation of WJ-MSCs was performed by explant technique on cultivation. The samples were washed three times in 3% P/S PBS. The umbilical cord was massaged to remove blood from the vessels. After this procedure, a longitudinal incision was made along the umbilical vein to remove the blood vessels from the tissue (Supplementary Figure S1).

The umbilical cord was divided into fragments placed in 75 cm2 flasks (23–25 per flask) and incubated in a 37 °C, 5% CO_2_ humidified incubator for 5 min. Then, 15 mL of complete medium (DMEM/F12) (Sigma-Aldrich, St. Louis, MO, USA) supplemented with 10% Fetal Bovine Serum (FBS) (Gibco®, Thermo Fisher®, Waltham, Massachusetts, USA) and 1% antibiotic (100 IU/mL penicillin, 0.1 mg/mL streptomycin - Thermo Fisher®, Waltham, Massachusetts, USA) (1% P/S) was added to each flask and incubated at 37 °C, 5% CO_2_. The first medium change occurred after five days and, subsequently, every 72 h until reaching 85% confluence [27,29].

### 5.6. Characterization of WJ-MSCs

#### 5.6.1. Flow Cytometry

Following trypsinization, a minimum of 1 × 10^6^ cells were suspended in 1 mL of PBS with 5% human albumin (HA) (Sigma-Aldrich^®^ in St. Louis, MO, USA). From this carefully prepared suspension, 200 μL was placed into four cytometry tubes, and the appropriate antibodies conjugated following to the manufacturer were added in accordance with Supplemental Table S1. The cell suspension was divided into four cytometry tubes, and conjugated antibodies were added in accordance with Table 3. After this, 400 μL of PBS was added to the tubes and centrifuged at 300× *g* for 5 min. The supernatant was discarded, and the pellet was resuspended in 400 μL of PBS, followed by the addition of 10 μL of 7-AAD. Finally, the samples were processed using a cytometer and analyzed with Infinicyt Flow Cytometry software, Version 1.6.0 (histograms are shown in Supplementary Figure S1) for the gating strategy, which excluded non-viable cells (those positive for the 7-AAD marker) and compared each CD marker with the isotype control. Markings overlapping with the isotype control were considered negative for the analyzed marker, while those not overlapping or positioned to the left of the isotype control were considered positive. The used cytometer was FACS Calibur (Becton Dickinson, Franklin Lakes, NJ, USA). The histograms represent the cytometry of the WJ-MSC for the markers CD13, CD90, CD105, CD34, CD73, CD45, and HLA-DR in Supplementary Figure S2.

#### 5.6.2. Trilineage Assay

When WJ-MS cells reached 85% confluence, the culture medium was supplemented with 0.5 µM dexamethasone, 0.5 mM isobutyl-methylxanthine, and 50 µM indomethacin (Sigma-Aldrich^®^ in St. Louis, MO, USA), for adipogenic differentiation. The cells were cultured in the differentiation medium for 14 days, with the medium being changed twice a week. The accumulation of lipid vesicles was detected through oil red O staining (Sigma-Aldrich^®^, St. Louis, MO, USA).

For the osteogenic and chondrogenic differentiations, the StemPro^®^ Osteogenesis Differentiation Kit and StemPro^®^ Chondrogenesis Differentiation Kit, respectively, were used following the manufacturer’s specifications (Thermo^®^, Waltham, MA, USA). The osteoblasts evaluated mineralization by staining the cells with alizarin red (Sigma-Aldrich^®^, St. Louis, MO, USA). At the same time, the production of proteoglycans in the chondroblasts was detected through staining with alcian blue.

### 5.7. Differentiation of Neural Precursors

The neural precursors were produced from WJ-MSCs. The first step was the formation of neurospheres by seeding the WJ-MSCs on the NFBX matrix (natural functional biopolymer matrix), as outlined by Stricker et al., 2021 and Dobuchak et al., 2022 [29,33]. After 24 h, the complete medium (DMEM/F12 supplemented with 10% FBS and 1% P/S) was changed, and the plates were incubated at 37 °C with 5% CO_2_. Then, the WJ-MSCs were seeded into the membrane at a concentration 1 × 10^4^ in 20 μL of the complete medium and incubated in a 37 °C, 5% CO_2_ incubator for 20 min. Then, a complete medium for cell culture was added and changed twice a week until neurospheres were produced.

Neurospheres were used to expand neural precursors; they were removed from the wells using a 100 μL micropipette and transferred to a 15 mL tube containing 5 mL of complete medium. They were then centrifuged at 300× *g* for 10 min. After centrifugation, the supernatant was discarded, and 2 mL of trypsin was added to the Falcon tube, which was incubated in an incubator (37 °C, 5% CO_2_) for 5 min. After incubation, 2 mL of complete medium was added to the tube, followed by another centrifugation at 300× *g* for 10 min. After centrifugation, the supernatant was discarded, the pellet was resuspended in 5 mL of complete medium, the cells were counted, and they were seeded in 75 cm^2^ flasks at a concentration of 1 × 10^4^/cm with 20 mL of medium. The flasks were then incubated at 37 °C with 5% CO_2_, and the medium was changed twice a week until the confluence was 85% reached [29,33].

### 5.8. Immunocytochemistry of Neural Precursors

For all immunocytochemical analyses, the cells were subjected to three washes with PBS and then fixed with 4% paraformaldehyde (Sigma-Aldrich^®^, St. Louis, MO, USA) for 20 min at room temperature. After fixation, another round of PBS washes was performed. The cells were permeated using a PBS solution containing 3% Triton X-100 (Sigma-Aldrich^®^, St. Louis, MO, USA) and 10% FBS for 5 min at room temperature. Subsequently, the cells were washed with PBS. The plates were then incubated overnight at 4 °C with the primary antibodies. Following this incubation, the plates were washed with PBS and incubated with the FITC-conjugated or Cy5-conjugated secondary antibodies. Finally, the plates were examined using an inverted fluorescence microscope (Axio Vert A1, Carl Zeiss, Oberkochen, Germany).

### 5.9. Cell Therapy

After 14 days of EAE model induction, the animals underwent the cell therapy procedure. Cell therapy was performed by intracerebral ventricular injection. For the transplantation, the animals were anesthetized with combined ketamine and xylazine (Syntec^®^, Tamboré, SP, Brazil) via intraperitoneal and placed in a ventral position [47]. The animal head was thricomized, and the antiseptic iodine alcohol solution was added to the upper area (Rioquímica^®^, São José do Rio Preto, São Paulo, Brazil). The skull was fixed to the stereotaxic apparatus using ear bars inserted into the external auditory meatus to prevent head movement. A longitudinal and median incision was made on the skin using n.20 surgical blades after topical iodine antiseptic preparation. Dissection was performed subperiosteally with the assistance of a periosteal elevator to expose the cranial vault adequately, focusing on the sutures, by making a dorsal dissection in the midline region between the frontal bones and the supraorbital process. The selected trepanation hole was created using a motor-driven drill with a 1/8-inch burr, following the Paxinos and Watson stereotaxic atlas for rats (2013) with the following coordinates: −0.09 mm posterior (from the Bregma), 1.4 mm from the midline (sagittal suture), taking care not to damage the cerebral cortex with the drill tip [48].

The Hamilton syringe containing 1 × 10^5^ cells in 10 uL of physiological solution (Fresenius^®^, Bad Homburg, Germany) was fixed to the stereotaxic apparatus (Sciencelabor, Ribeirão Preto, São Paulo, Brazil). The content was slowly injected following the defined coordinates of the trepanation site and reached a depth of 3.4 mm to reach the cerebral ventricles, as outlined by Paxinos and Watson, 2013 [48]. After withdrawing the needle, we sealed the trepanation hole to prevent reflux of the injected cells and sutured the skin incision with simple interrupted sutures.

### 5.10. Histopathology and Immunohistochemistry (IHC) Analysis

Ten days after treatment, the animals were anesthetized, euthanized, and perfused by cannulation of the left ventricle with PBS, pH 7.2, for 2 min, followed by PBS, pH 7.2, containing 4% paraformaldehyde (Dinâmica Química Contemporânea LTDA, São Paulo, SP, Brazil). After perfusion, the brain was collected and stored in a 10% paraformaldehyde solution for histopathological and immunohistochemical analysis.

For the histopathological analysis, the samples were dehydrated using alcohol at 70%, 80%, 90%, and 100%, cleared in xylene (Dinâmica Química Contemporânea LTDA, São Paulo, SP, Brazil), and embedded in paraffin (Êxodo Científica, Sumaré, SP, Brazil). The blocks were sectioned into 5 µm slices for histochemical techniques using Hematoxylin and Eosin (H&E) (NewProv^®^, Pinhais, PR, Brazil; Eosin: BIOTEC Analytical Reagents^®^, Lages, SC, Brazil) and Luxol Fast Blue (Sigma-Aldrich^®^, St. Louis, MO, USA) staining methods.

Brain tissue with H&E was used to analyze the neuroinflammation findings, including perivascular leukocyte infiltration, scattered leukocytes throughout the parenchyma, microglial activation, astrocyte activation, and cell death, indicated by the appearance of pyknotic neurons. Luxol slides were analyzed using Image-Pro Plus 4.5 (Media Cybernetics, Rockville, MD, USA) software to quantify the myelin concentration.

The analysis was in the frequency and percentage of occurrence of leukocyte infiltration around blood vessels (cuffing), leukocytes in the parenchyma, and activation of microglia, astrocytes, and cell death.

In the IHC analysis, markers of interest such as CD4, CD8, IL-4, TNFα, and INFγ were identified using anti-CD4, anti-CD8, anti-IL-4, anti-TNFα, and anti-INFγ antibodies (Sigma-Aldrich^®^, St. Louis, MO, USA). The secondary antibody was the Reveal Polyvalent HRP-DAB Detection System (Spring Bioscience, Pleasanton, CA, USA). Slides were scanned using the Axio Scan.Z1 scanner (Carl Zeiss, Oberkochen, Germany). Then, the ZEN 3.1 blue edition software (Carl Zeiss, Oberkochen, Germany) was employed to generate ten images of random fields (40× objective) for each sample and each interleukin. Image-Pro Plus 4.5 software (Media Cybernetics, Rockville, MD, USA) was used to quantify the areas of immunopositivity for the IL-4, TNFα, and INFγ markers. CD4 and CD8 cells were counted from the ten randomly produced field images, and, for each sample, the mean was established for statistical analysis.

The histopathological analysis was performed to counter the frequency of the findings that were used for finding the immunopositivity of IL-4, TNFα, INFγ, CD4, and CD8 with % expression; for inflammatory infiltrates, it was classified by their presence: perivascular leukocytic and parenchyma infiltrates: mild (+), moderate (++), and severe (+++).

### 5.11. Statistical Analysis

After data collection, the data were registered into Excel. Descriptive statistical analysis was performed using calculations of central tendency and dispersion measures, as well as frequency counts. For inferential statistical analysis of quantitative variables, the Anderson–Darling test was employed to check for data normality, with *p* < 0.01 indicating normal distribution. Subsequently, the ANOVA and Tukey tests were used for parametric variables, while Kruskal–Wallis and Dunn tests were applied for non-parametric variables. Statistical significance was considered when *p* < 0.05. Pearson’s Chi-squared test was conducted for binary variables, and statistically associated variables had *p* < 0.05. Stata18 and Minitab18 software were used for these analyses.

## Figures and Tables

**Table 1:** Comparisons of weights (g) between animal groups, with *p* < 0.05 representing a statistically significant difference at a 95% CI.

Weight (g)	*p*-Value
Non-Induced vs. Sham	0.011
Non-Induced vs. MSCs	0.001
Non-Induced vs. NPs	0.000
Non-Induced vs. MSCs + NPs	0.000
Sham vs. MSCs	0.363
Sham vs. NPs	0.001
Sham vs. MSCs + NPs	0.002
MSCs vs. NPs	0.000
MSCs vs. MSCs + NPs	0.000
NPs vs. MSCs + NPs	0.000

**Table 2:** Results of CD4/CD8 ratio ≤1.0 with histopathological findings. *p* > 0.05 was considered statistically significant at the 95% confidence interval.

CD4/CD8 ≤ 1.0 vs. Perivascular Infiltrates		CD4/CD8 ≤ 1.0 vs. Parenchyma Infiltrates		CD4/CD8 ≤ 1.0 vs. Cell Activation and Death	
Non-induced	*p*	Non-induced	*p*	Non-induced	*p*
Mild (+)	0.030	Mild (+)	0.022	Microglia	0.133
Moderate (++)	0.035	Moderate (++)	0.041	Astrocyte	0.231
Severe (+++)	0.346	Severe (+++)	0.046	Cell death	0.255
Sham	*p*	Sham	*p*	Sham	*p*
Mild (+)	0.257	Mild (+)	0.022	Microglia	0.011
Moderate (++)	0.379	Moderate (++)	0.017	Astrocyte	0.025
Severe (+++)	0.455	Severe (+++)	0.012	Cell death	0.270
WJ-MSC	*p*	WJ-MSC	*p*	WJ-MSC	*p*
Mild (+)	0.222	Mild (+)	0.350	Microglia	0.366
Moderate (++)	0.235	Moderate (++)	0.466	Astrocyte	0.247
Severe (+++)	0.044	Severe (+++)	0.688	Cell death	0.577
NP	*p*	NP	*p*	NP	*p*
Mild (+)	0.034	Mild (+)	0.112	Microglia	0.038
Moderate (++)	0.047	Moderate (++)	0.024	Astrocyte	0.333
Severe (+++)	0.049	Severe (+++)	0.042	Cell death	0.421
WJ-MSC+NP	*p*	WJ-MSC+NP	*p*	WJ-MSC+NP	*p*
Mild (+)	0.012	Mild (+)	0.242	Microglia	0.455
Moderate (++)	0.037	Moderate (++)	0.445	Astrocyte	0.587
Severe (+++)	0.299	Severe (+++)	0.032	Cell death	0.592

Non-induced group (N-I), Wharton’s jelly mesenchymal stem cells (WJ-MSCs), and neural precursors (NPs).

**Table 3:** Flow cytometry panel design.

Tube	Content
1	Cells without markers
2	Isotypic control
3	CD90 FITC/CD105 PE/7-AAD PERCP/CD34 PE-CY7/CD73 APC/CD45 APC-CY7
4	HLA-DR FITC/CD13 PE/7-AAD PERCP/CD34 PE-CY7/CD45 APC-CY7
FMO	CD105 PE/7-AAD PERCP/CD34 PE-CY7/CD73APC/CD45 APC-CY7
FMO	CD90 FITC/7-AAD PERCP/CD34 PE-CY7/CD73 APC/CD45 APC-CY7

Fluorescence Minus One (FMO).

## Data Availability

Data are contained within the article and Supplementary Materials.

## Supplementary Materials

The following supporting information can be downloaded at: https://www.mdpi.com/article/10.3390/ijms25136996/s1.
