# Supplementary material for: Cellular Therapy in Experimental Autoimmune Encephalomyelitis as an Adjuvant Treatment to Translate for Multiple Sclerosis
Source: Int J Mol Sci. 2024 Jun 26;25(13):6996. doi: 10.3390/ijms25136996 (PMC11241124; doi:10.3390/ijms25136996)
Supplement: Supplementary file 1 [file ijms-25-06996-s001.zip › ijms-3011135-supplementary.pdf]

Cellular Therapy in Experimental Autoimmune Encephalomyelitis as Adjuvant Treatment to Translate for Multiple Sclerosis.

Maiara Carolina Perussolo[1], Bassam Felipe Mogharbel[1], Cláudia Sayuri Saçaki[1], Nádia Nascimento da Rosa[1], Ana Carolina Irioda[1], Nathalia Barth de Oliveira[1], Julia Maurer Appel[1], Larissa Lührs[1], Leanderson Franco Meira[2], Luiz Cesar Guarita-Souza[2], Seigo Nagashima[3], Caroline Busatta Vaz de Paula[3], Lucia de Noronha[3], Idiberto José Zotarelli-Filho[4], Eltyeb Abdelwahid[5], Katherine Athayde Teixeira de Carvalho[1*].

Appendix material

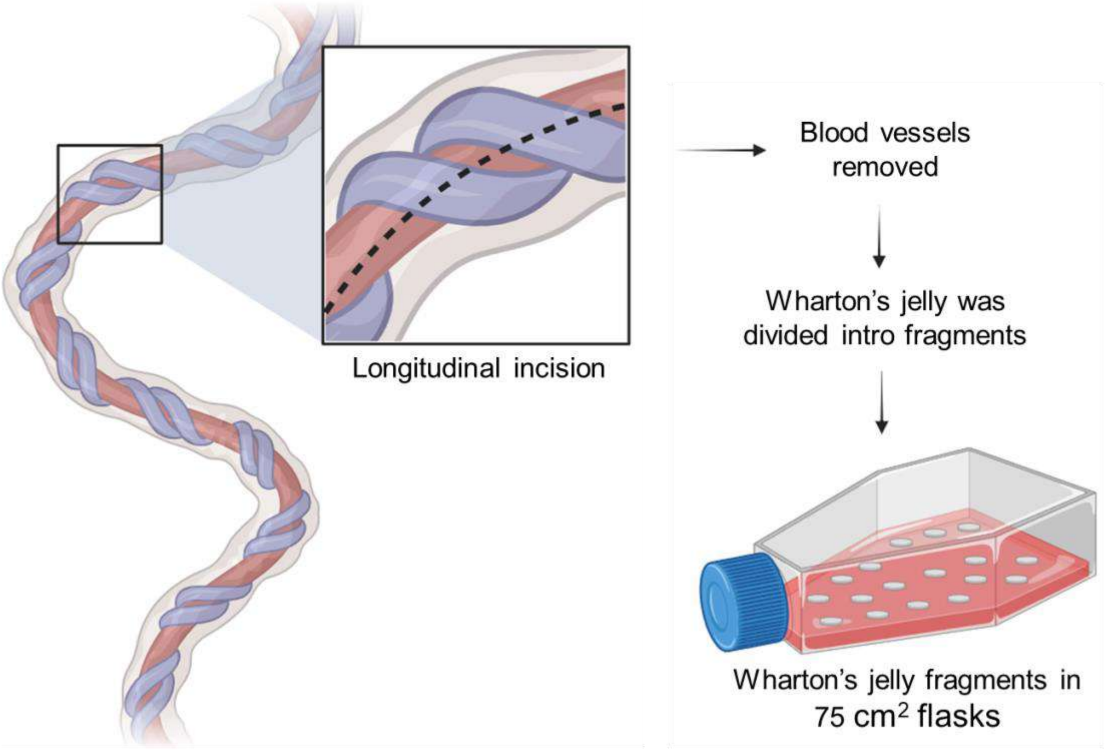

Supplementary Figure S1. Explant technique for WJ-MSC isolation. PBS was used to wash the collected umbilical cord, and the vessels were removed. Following this, fragments of the cord were placed in 75 cm^2 flasks. Created with BioRender.

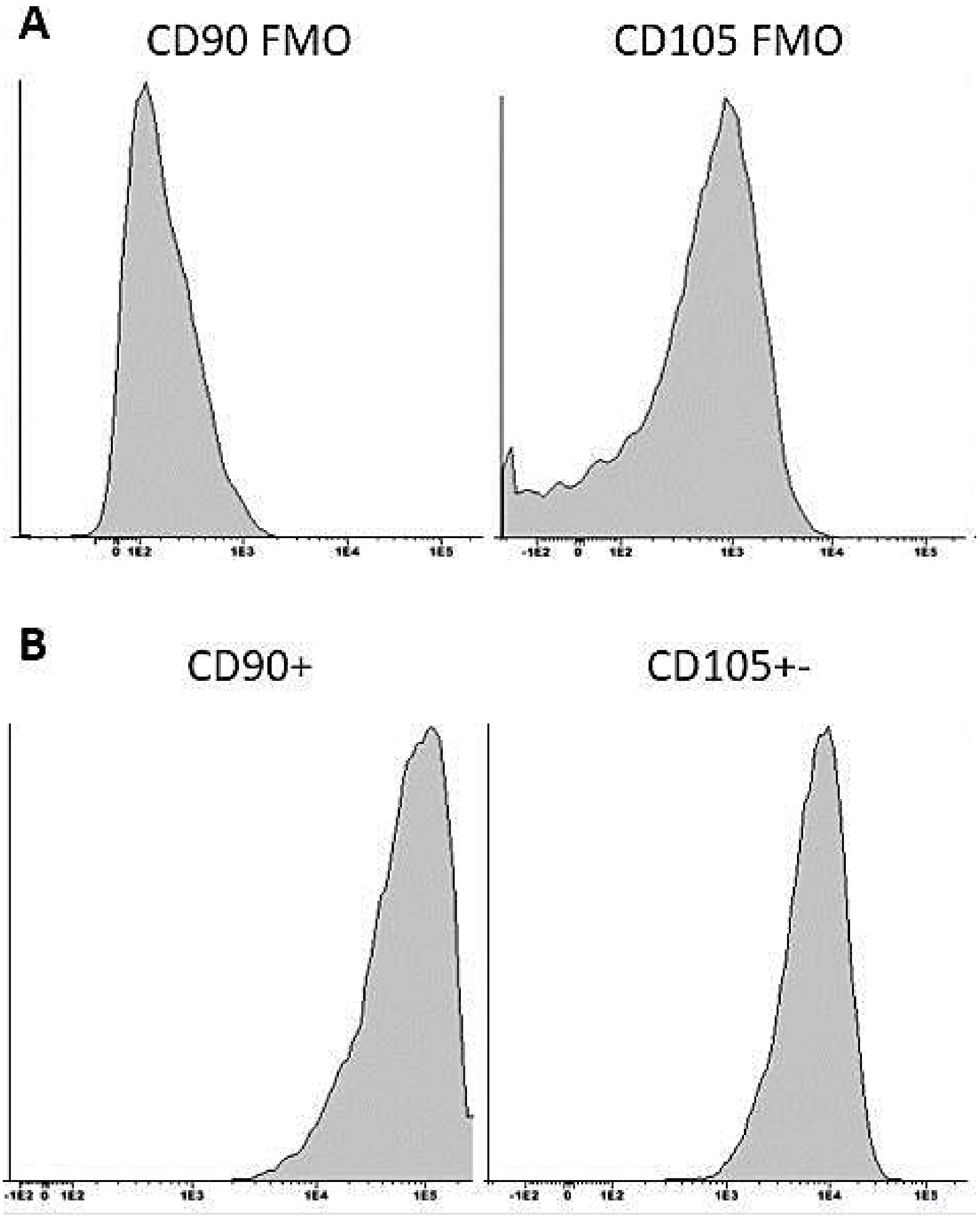

Supplementary Figure S2. Flow cytometry histograms. (A) FMO of the markers CD90, and CD105; (B) Flow cytometry of WJ-MSC showing positive staining for the same markers.

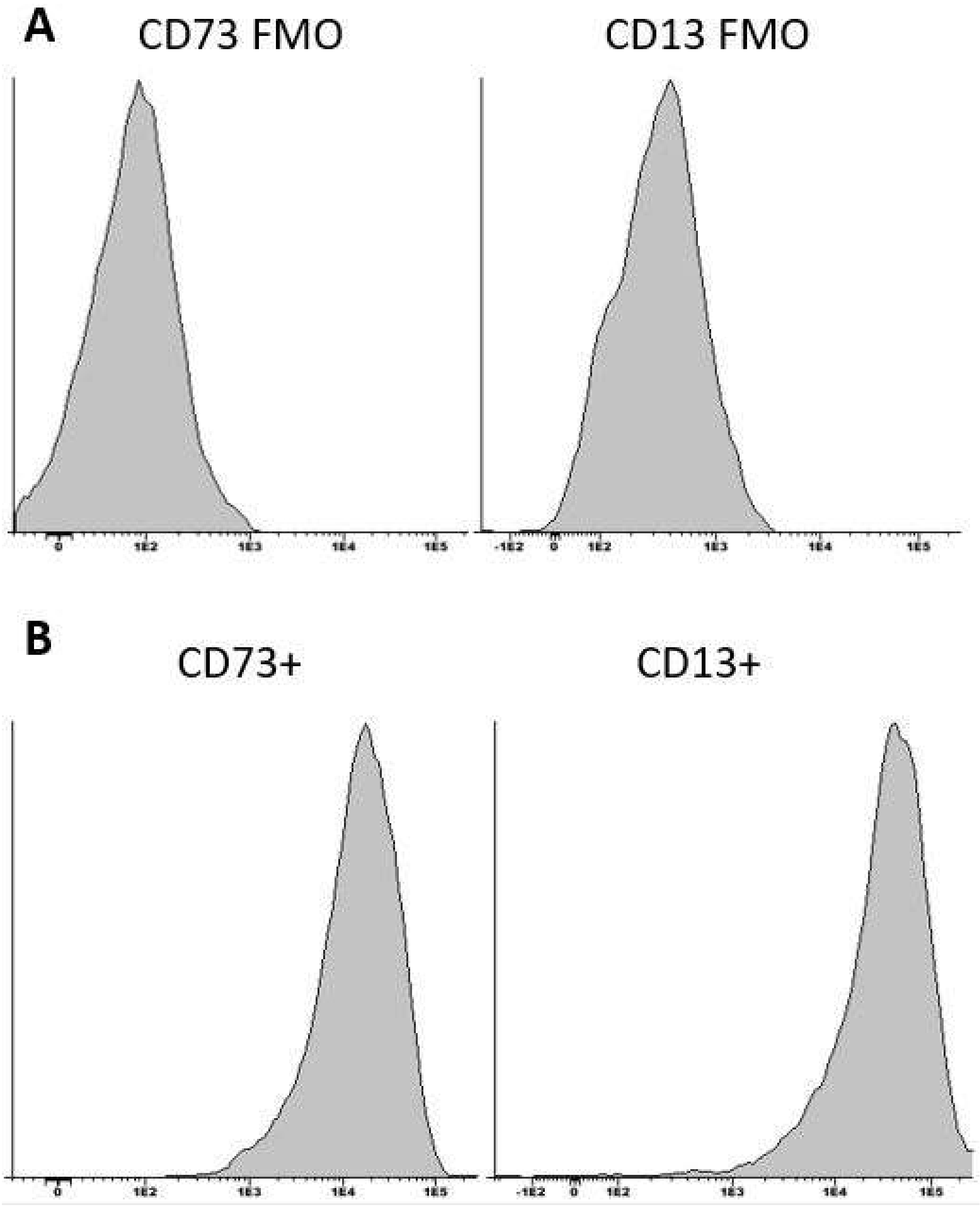

Supplementary Figure S3. Flow cytometry histograms. (A) FMO of the markers CD73, and CD13; (B) Flow cytometry of WJ-MSC showing positive staining for the same markers.

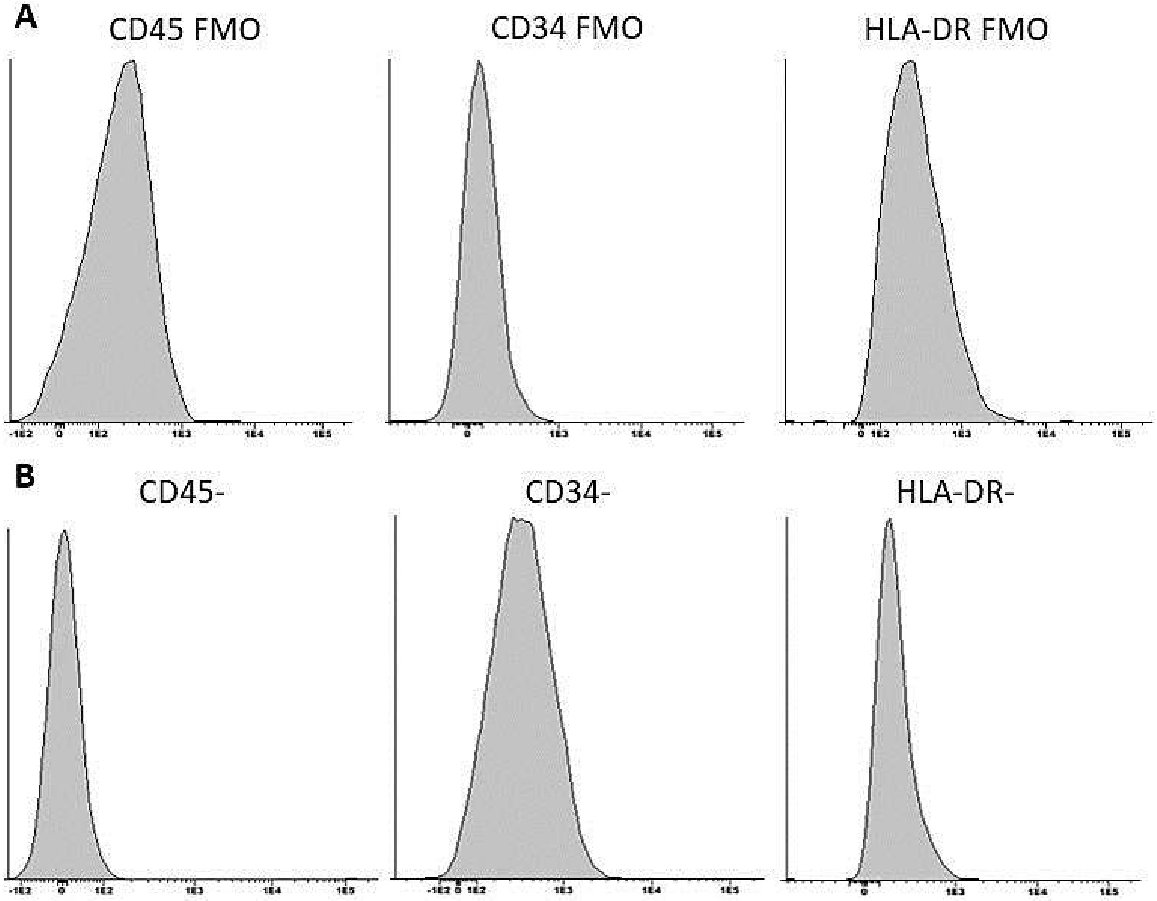

Supplementary Figure S4. Flow cytometry histograms. (A) FMO of the markers CD45, CD34, and HLA-DR; (B) Flow cytometry of WJ-MSC showing negative staining for the same markers.

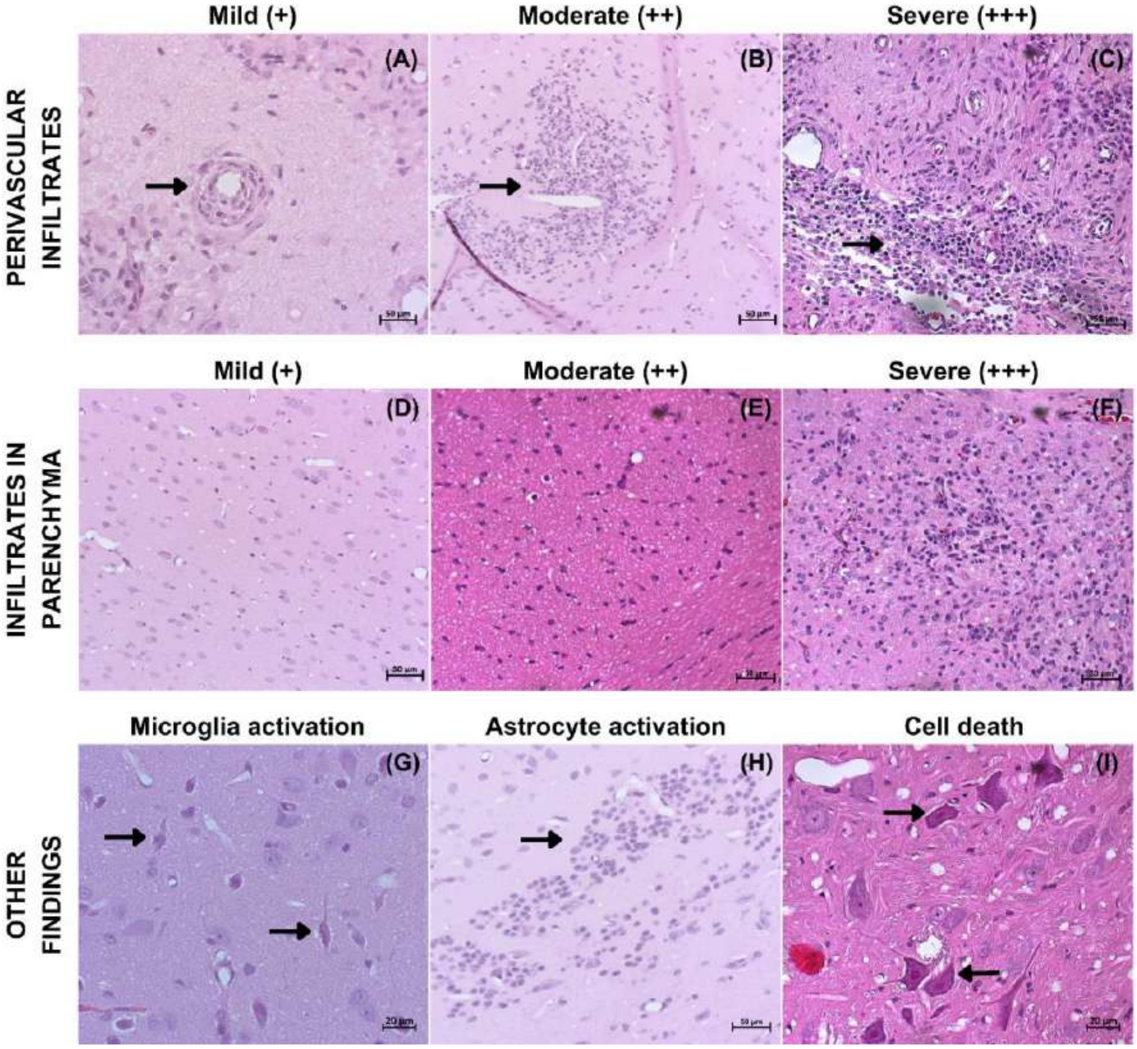

Supplementary Figure S5. Histopathological findings for neuroinflammation analysis. Considered were perivascular leukocyte infiltration as mild (A), moderate (B), and severe (C); Presence of parenchymal leukocyte infiltration as mild (D), moderate (E), and severe (F); Microglial activation (G); Astrocytic activation (H); and cell death (I), highlighted by the presence of pyknotic neurons. Images were captured using optical microscopy (Carl Zeiss Microscopy, Zeiss, Jena, Germany).

Supplementary Table S1. *Flow Cytometry Markers*

Antibody	*Volume (µL)*	*Clone*	*Fluorochrome*	*Manufacturer*
CD34	***3***	***581***	***PE-Cy7***	***BD® Biosciences, CA, USA***
CD45	***3***	***2D1***	***APC-Cy7***	***BD® Biosciences, CA, USA***
CD73	***3***	***AD2***	***APC***	***BD® Biosciences, CA, USA***
CD90	***3***	***5E10***	***FITC***	***Biolegend®, CA, USA***
CD105	***3***	***266***	***PE***	***BD® Biosciences, CA, USA***
CD13	***5***	***WM15***	***PE***	***IO Test – Beckman Coulter®, CA, USA***
HLA-DR	***5***	***L243***	***FITC***	***BD® Biosciences, CA, USA***

Supplementary Graphic S1. Predictive binary logistic regression analysis of animals in the MSC group vs. Sham, with p>0.05 with a statistical difference in the 95% CI.

Regression for Sham vs MSC
Prediction Report

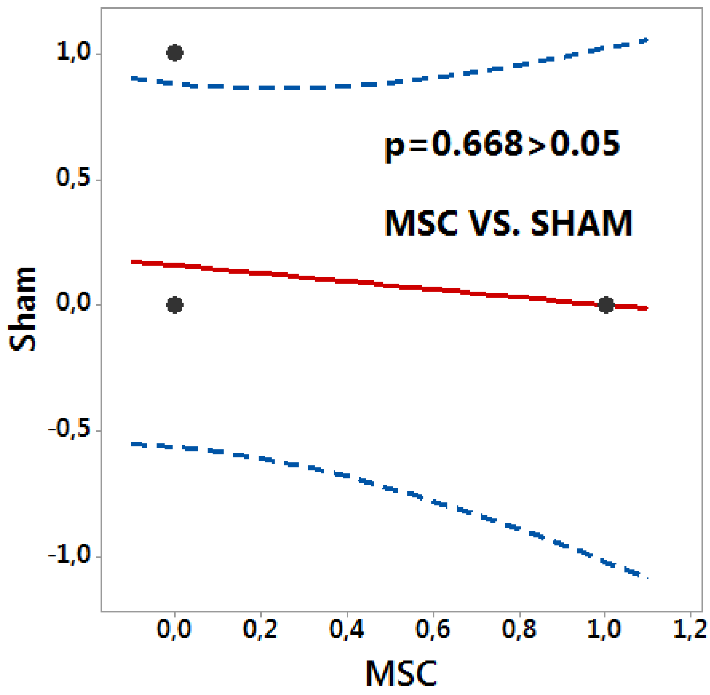

X	Predicted Y	95% PI
-0,1	0,17310	(-0,55266; 0,89885)
-0,05	0,16523	(-0,55765; 0,88810)
0	0,15736	(-0,56443; 0,87915)
0,05	0,14949	(-0,57302; 0,87200)
0,1	0,14162	(-0,58340; 0,86665)
0,15	0,13376	(-0,59557; 0,86308)
0,2	0,12589	(-0,60948; 0,86126)
0,25	0,11802	(-0,62510; 0,86114)
0,3	0,11015	(-0,64237; 0,86268)
0,35	0,10228	(-0,66123; 0,86580)
0,4	0,094416	(-0,68162; 0,87045)
0,45	0,086548	(-0,70346; 0,87656)
0,5	0,078680	(-0,72667; 0,88403)
0,55	0,070812	(-0,75119; 0,89281)
0,6	0,062944	(-0,77692; 0,90281)
0,65	0,055076	(-0,80380; 0,91396)
0,7	0,047208	(-0,83176; 0,92617)
0,75	0,039340	(-0,86071; 0,93939)
0,8	0,031472	(-0,89059; 0,95354)
0,85	0,023604	(-0,92134; 0,96855)
0,9	0,015736	(-0,95289; 0,98436)
0,95	0,007868	(-0,98519; 1,0009)
1	0	(-1,0182; 1,0182)
1,05	-0,00787	(-1,0518; 1,0361)

Source: Own Authorship.

Supplementary Graphic S2. Predictive binary logistic regression analysis of animals in the Sham vs. NP, with p>0.05 with a statistical difference in the 95% CI.

Regression for Sham vs NP
Prediction Report

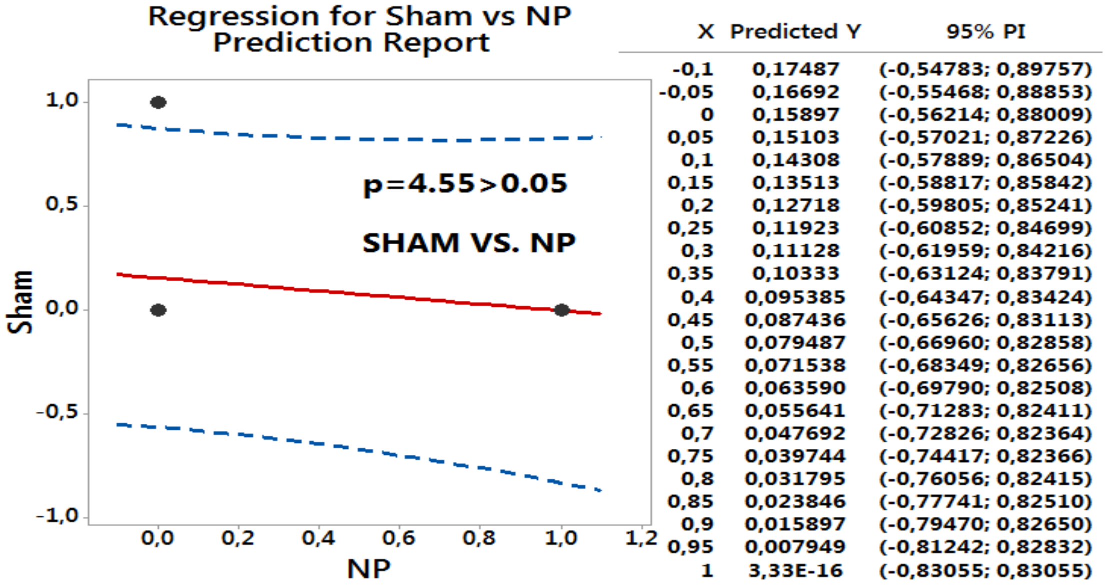

X	Predicted Y	95% PI
-0,1	0,17487	(-0,54783; 0,89757)
-0,05	0,16692	(-0,55468; 0,88853)
0	0,15897	(-0,56214; 0,88009)
0,05	0,15103	(-0,57021; 0,87226)
0,1	0,14308	(-0,57889; 0,86504)
0,15	0,13513	(-0,58817; 0,85842)
0,2	0,12718	(-0,59805; 0,85241)
0,25	0,11923	(-0,60852; 0,84699)
0,3	0,11128	(-0,61959; 0,84216)
0,35	0,10333	(-0,63124; 0,83791)
0,4	0,095385	(-0,64347; 0,83424)
0,45	0,087436	(-0,65626; 0,83113)
0,5	0,079487	(-0,66960; 0,82858)
0,55	0,071538	(-0,68349; 0,82656)
0,6	0,063590	(-0,69790; 0,82508)
0,65	0,055641	(-0,71283; 0,82411)
0,7	0,047692	(-0,72826; 0,82364)
0,75	0,039744	(-0,74417; 0,82366)
0,8	0,031795	(-0,76056; 0,82415)
0,85	0,023846	(-0,77741; 0,82510)
0,9	0,015897	(-0,79470; 0,82650)
0,95	0,007949	(-0,81242; 0,82832)
1	3,33E-16	(-0,83055; 0,83055)
1,05	-0,00795	(-0,84907; 0,83318)

Source: Own Authorship.

Supplementary Graphic S3. Predictive binary logistic regression analysis of animals in the MSC + NP vs. Sham, with p>0.05 with a statistical difference in the 95% CI.

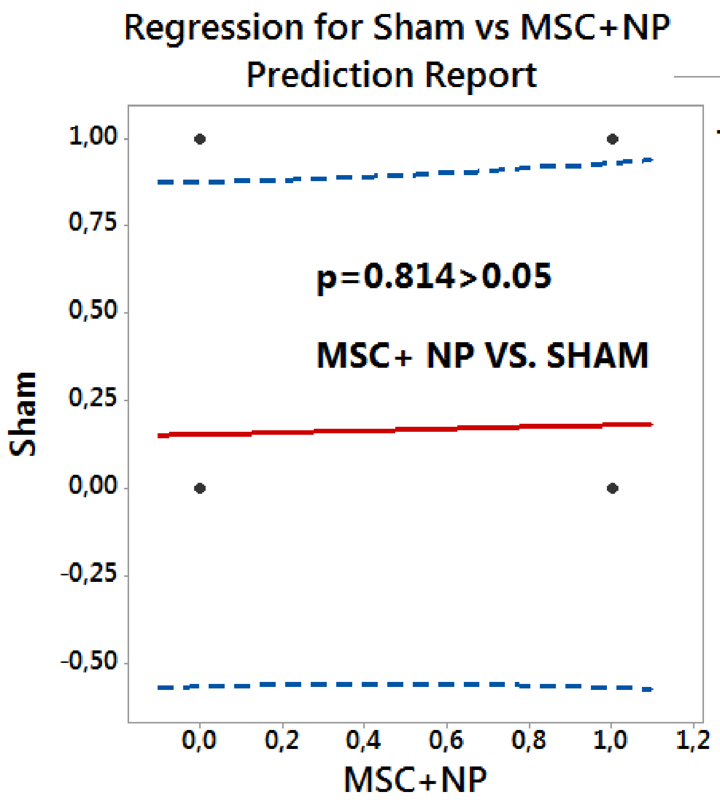

X	Predicted Y	95% PI
-0,1	0,15241	(-0,57045; 0,87526)
-0,05	0,15374	(-0,56866; 0,87615)
0	0,15508	(-0,56705; 0,87721)
0,05	0,15642	(-0,56560; 0,87844)
0,1	0,15775	(-0,56433; 0,87984)
0,15	0,15909	(-0,56324; 0,88142)
0,2	0,16043	(-0,56231; 0,88317)
0,25	0,16176	(-0,56156; 0,88509)
0,3	0,16310	(-0,56098; 0,88718)
0,35	0,16444	(-0,56057; 0,88945)
0,4	0,16578	(-0,56033; 0,89189)
0,45	0,16711	(-0,56027; 0,89449)
0,5	0,16845	(-0,56037; 0,89727)
0,55	0,16979	(-0,56064; 0,90021)
0,6	0,17112	(-0,56107; 0,90332)
0,65	0,17246	(-0,56168; 0,90659)
0,7	0,17380	(-0,56244; 0,91004)
0,75	0,17513	(-0,56337; 0,91364)
0,8	0,17647	(-0,56446; 0,91740)
0,85	0,17781	(-0,56571; 0,92133)
0,9	0,17914	(-0,56712; 0,92541)
0,95	0,18048	(-0,56869; 0,92965)
1	0,18182	(-0,57041; 0,93405)
1,05	0,18316	(-0,57228; 0,93859)

Source: Own Authorship.
